# Complement-mediated ADCP as a distinct and finite cytotoxic mechanism of monoclonal antibodies

**DOI:** 10.3389/fimmu.2026.1788948

**Published:** 2026-04-13

**Authors:** Carly M. Van Wagoner, Jonathan J. Pinney, Hanna M. Sovinski, Brett P. Ransegnola, Nydia Jaimes-Delgadillo, Charles C. Chu, Clive S. Zent, Michael R. Elliott

**Affiliations:** 1 Department of Microbiology, Immunology, and Cancer Biology, University of Virginia, Charlottesville, VA, United States; 2 Department of Pathology, University of Virginia, Charlottesville, VA, United States; 3 Beirne B. Carter Center for Immunology Research, University of Virginia, Charlottesville, VA, United States; 4 Department of Medicine, University of Rochester Medical Center, Rochester, NY, United States; 5 Wilmot Cancer Institute, University of Rochester Medical Center, Rochester, NY, United States; 6 Department of Microbiology and Immunology, University of South Alabama, Mobile, AL, United States; 7 Mitchell Cancer Institute, University of South Alabama Health, Mobile, AL, United States

**Keywords:** immunotherapy, innate immunity, live-cell imaging, macrophages, monoclonal antibodies, phagocytosis

## Abstract

**Introduction:**

Macrophage phagocytosis is a major cytotoxic mechanism for therapeutic monoclonal antibodies (mAbs) that opsonize target cells. This antibody-dependent cellular phagocytosis (ADCP) can occur via the Fcγ or complement pathways, but the relative contribution of these pathways to mAb-mediated cell clearance is not known. Here, we analyzed the kinetics, functional cooperation, and phagocytic capacities of Fcγ receptor-dependent and complement-dependent ADCP, separately and concomitantly, in primary macrophages challenged with mAb-opsonized lymphocytes.

**Methods:**

Using quantitative live-cell imaging of primary mouse macrophages, genetic disruption of Fcγ receptor signaling, and controlled modulation of complement activity, we directly compared the kinetics, capacity, and exhaustion behavior of ADCP via the Fcγ (fADCP) and complement (cADCP) pathways.

**Results:**

cADCP operates as a mechanistically independent phagocytic pathway with distinct temporal dynamics. Relative to fADCP, cADCP exhibits delayed onset but substantially greater cumulative target clearance. When both pathways are engaged simultaneously, their effects on target removal are additive, indicating functional non-redundancy. Notably, macrophages rendered refractory to further phagocytosis following fADCP retain full capacity for cADCP, demonstrating that complement receptor–mediated engulfment can bypass Fcγ receptor–associated hypophagia. However, despite its greater capacity, cADCP is also finite, as increasing target burden induces a dose-dependent state of complement-associated phagocytic exhaustion that is kinetically distinct from fADCP hypophagia and is largely reversible within 24 h.

**Discussion:**

Our mechanistic understanding of mAb-mediated cytotoxicity via ADCP is disproportionately focused on Fcγ receptor engagement and signaling, with comparatively less emphasis on the role of complement activation. These findings establish complement-mediated ADCP as a quantitatively powerful macrophage effector pathway that can be leveraged to enhance the overall cytotoxic efficacy of mAbs. Further, our work provides a functional framework for understanding how Fcγ receptor and complement pathways differentially contribute to macrophage cytotoxic capacity and highlights effector exhaustion as a shared but mechanistically distinct constraint on sustained antibody-mediated cell clearance. Effector exhaustion therefore represents a fundamental bottleneck to durable responses to ADCP-inducing therapeutic antibodies, but one that can potentially be mitigated via utilization of both ADCP pathways.

## 1 Introduction

Unconjugated monoclonal antibodies (mAb) that mediate target cell clearance through phagocytosis are widely used therapies in autoimmunity and cancer. However, we and others have recently shown that the therapeutic efficacy of such mAbs is limited by the finite phagocytic capacity of tissue resident macrophages (1–4). For mAbs targeting B cell surface antigens such as CD20, CD38, and CD52, therapeutic activity largely depends on engagement of the innate immune system through FcγR crosslinking, complement activation, or both (5–11). Antibody-dependent cellular phagocytosis (ADCP) by tissue-resident macrophages and antibody-dependent cellular cytotoxicity (ADCC) by NK cells are major cytotoxic effector mechanisms triggered by Fc gamma receptor (FcγR) engagement (12, 13); however, cell depletion studies have revealed that macrophages are the primary effectors of mAb driven antitumor activity and that ADCP is the chief mechanism of cell killing (11, 14–18). In particular, liver resident macrophages have been shown to drive the majority of B cell clearance during anti-CD20 immunotherapy in mice (7, 9, 10). However, we currently lack a strong understanding of the relative and potentially cooperative contributions of the two chief ADCP pathways, FcγR- (fADCP) and complement-dependent (cADCP), to the overall cell clearance activity of therapeutic mAbs. This knowledge gap largely reflects the reliance on commonly used anti-CD20 clones (e.g. 5D2) that are weak activators of mouse complement and thus largely drive cell clearance solely via fADCP (10).

Some therapeutic mAbs (e.g., rituximab, ofatumumab, alemtuzumab) activate complement, leading to complement-dependent cytotoxicity (CDC) and/or cADCP (19–26). Although CDC has been extensively investigated, the contribution of cADCP to therapeutic antibody activity remains poorly defined (27, 28). Studies in C1q-, C3-, or C4-deficient mice show that blocking steps within the classical complement pathway, including CDC, does not impair anti-CD20 mAb-mediated B-cell depletion *in vivo*, implying that FcγR-dependent ADCP (fADCP) is the dominant effector mechanism (17, 29–31). In contrast, other reports demonstrate that complement activation can enhance mAb therapy (32–34), and mAbs engineered for stronger complement fixation drive greater phagocytosis of opsonized targets (10, 35–40). These conflicting data highlight an important yet unproven contribution of cADCP to therapeutic mAb activity.

Although mAb immunotherapy has improved patient outcomes, it is rarely curative in part because of multiple mechanisms of therapeutic resistance. One such mechanism is the finite capacity of the immune system to execute the cytotoxic functions triggered by the therapeutic mAb (1, 3, 41, 42). The concept that phagocytes possess a finite capacity for ADCP is intuitively clear, yet only recently has this limitation been formally demonstrated and its cellular basis investigated. In 2016, Church et al. used co-cultures of human monocyte-derived macrophages and CLL cells to show that >90% of all ADCP induced by therapeutic anti-CD20 mAbs occurs within the first hour of treatment, with minimal additional CLL clearance seen over the next 23 hours (1). Building on this observation, Pinney et al. performed rechallenge experiments with primary mouse and human macrophages to directly assess the phagocytic capacity following an initial round of ADCP (3). After engulfing a single “meal” of mAb-opsonized target cells, macrophages exhibited a >90% reduction in phagocytic capacity upon rechallenge with fresh targets and mAb. Mechanistically, this loss of phagocytic activity correlated with a strong reduction in surface FcγRs expression both *in vitro* and *in vivo*. Subsequent studies have further defined novel aspects of phagocyte capacity and its relevance to cell clearance in efferocytosis and cancer (41, 43–45). Notably, however, these studies were employed in exclusively complement-deficient culture conditions, leaving the question of phagocytic exhaustion during cADCP unresolved (3).

In the present study, we employed *in vitro* approaches to study macrophage cADCP capacity and the relationship between complement- and FcγR-mediated ADCP. We show that complement- and FcγR-mediated ADCP function as distinct, independent pathways with different clearance kinetics, with cADCP exhibiting greater overall cell clearance capacity. When both pathways are engaged simultaneously, their effects are additive; however, like fADCP, the cADCP pathway can become exhausted.

## 2 Methods

### 2.1 Mice

Animal experiments were conducted in accordance with the guidelines and regulations of the University of Virginia and approved by the University of Virginia Animal Care and Use Committee. Mouse strains C57BL/6J (wild-type), and B6.129P2-*Fcer1g^tm1Rav^* (gamma chain deficient) were obtained from Jackson Labs (Bar Harbor, ME) or Taconic Biosciences (Germantown, NY). Thymocytes and bone marrow cells were isolated from male and female mice between 4–20 weeks of age. All mice were maintained on a C57BL/6J background, and a mixture of both female and male mice were used in these experiments.

### 2.2 Chemicals and cell culture reagents

Commercial reagents were obtained from the following suppliers: heat-inactivated FBS (HI-FBS, Atlanta Biologicals); normal mouse serum (Jackson ImmunoResearch); cell culture media (Gibco); Murine M-CSF (Biolegend); Cell Tracker Deep Red and 5-TAMRA-SE (Thermo Fisher Scientific); CypHer5E NHS Ester (Cytiva); Cobra Venom Factor (Quidel).

### 2.3 Antibody crosslinking

C5 antibody (clone BB5.1; Creative Biolabs) crosslinking to protein g sepharose beads (Cytiva) was performed following crosslinking protocol published in Abcam protocol book (46), and crosslinking efficiency was evaluated by Coomassie blue stain.

### 2.4 Complement depletion

Three methods were used to deplete complement from mouse serum. Heat inactivation was done by incubating NMS in a 56° water bath for 60 minutes. CVF treatment followed manufacturer instructions. Briefly, NMS was incubated in a 37° water bath for 90 minutes with 10 units/mL of CVF (Quidel). Finally, C5 depletion followed the antibody crosslinking protocol. C5-crosslinked beads were incubated with NMS at 4 °C for 1 hour with gentle rotation prior to use in microscopy assays. NMS incubated with protein g Sepharose beads was used as a control. Immediately after the 1-hour incubation, beads were pelleted, and flowthrough NMS was collected for a C5 ELISA. Remaining NMS was stored at -20 °C until use for microscopy assays.

### 2.5 Enzyme-linked immunosorbent assay

ELISA for C5 (abcam, ab264609) was performed according to the instructions of the manufacturer.

### 2.6 Functional antibodies for ADCP assays

mAbs used in ADCP assays were toxin-free, cell culture grade, and were obtained from the following sources: BioLegend: anti-mouse CD90.2 (30-H12) and anti-mouse CD20 (QA18A73); Genentech: anti-mouse CD20 (5D2); InvivoGen: anti-mouse CD20 (18B12).

### 2.7 Antibodies for flow cytometry

Fluorescently-conjugated antibodies for flow cytometry were obtained from the following sources: BioLegend: anti-mouse CD64 (X54-5/7.1), anti-mouse CD16.2 (9E9), anti-mouse CD16 (S17014E), anti-mouse CD59a (mCD59.3), anti-mouse/human CD11b (M1/70), anti-mouse CD102 (AF647), anti-mouse CD11c (N418), anti-mouse F4/80 (BM8); Invitrogen: anti-mouse CD32b (AT130-2), anti-mouse VSIG4 (NLA14).

### 2.8 Primary cell isolation and culture

Mouse BMDM were derived from bone marrow isolated from the femurs of 5-20-week-old C57BL/6J and B6.129P2-*Fcer1g^tm1Rav^* mice and cultured in RFHP10 media (RPMI 1640, 10% HI-FBS, 10 mM HEPES, 1% penicillin-streptomycin-L-glutamine) supplemented with 20 ng/mL M-CSF for 6–10 days as previously described (47). Murine thymocytes were isolated from C57BL/6J mice as previously described (48).

### 2.9 Phagocytosis imaging assays

BMDM (day 6-10) were collected by trypsinization and seeded in 1 mL RFHP10 at 5x10^4^ cells per well of a 24-well treated tissue culture plate (Falcon) and cultured 18–24 hours. For ADCP assays lasting longer than 24 hours, 20 ng/mL M-CSF was added to RFHP10 at the time of reseeding to maintain BMDM viability throughout the experiment. Prior to ADCP assay, BMDM were stained with 1 μM Cell Tracker Deep Red for 10 min or 2.5 μM 5-TAMRA-SE for 20 min at 37°C in DPBS. BMDM were then washed with RFHP10 and allowed to recover for 2–4 hours prior to addition of target cells ± 10% normal mouse serum (NMS) and the plate centrifuged at 100xG for 1 min to sediment target cells. In some experiments, target cells were labeled with the pH sensitive dye cypHer5E (1 μM) in cation-free HBSS for 20 min prior to addition to BMDM. The plate was then mounted on a stage-top environmental chamber of a Nikon Ti-Eclipse inverted microscope to maintain 37°C/5% CO_2_ throughout the experiment. Phase contrast and fluorescent images were captured using a 20x objective at 2 min intervals for 3 loops before addition of mAb. Following addition of mAb, BMDM were imaged for 4–14 hours using the same microscope settings. For phagocytosis re-challenge assays, at indicated times after initial co-culture, wells were washed three times with RFHP10 to remove unengulfed cells and fresh cells and antibody added at indicated times in 1 mL RFHP and imaged as described above (49).

### 2.10 Phagocytic index calculations

Phagocytic indexes were calculated using image analysis features in the Nikon NIS-Elements software, as previously described (3, 49). Briefly, the number of macrophages in each frame was calculated using a binary layer detection method based on the fluorescence of the macrophage dye. Engulfed targets, which appear as circular dye voids within dye-labeled macrophages, were enumerated for each frame. The phagocytosis index was calculated by subtracting the average number of voids from the initial three frames of the experiment before antibody addition from raw void count in each subsequent frame collected. The initial background subtracted void count for each frame was then normalized to 100 macrophages. In rechallenge assays the index was re-zeroed at time of re-challenge by subtracting the number of voids from the initial re-challenge frame from all proceeding image loops. Quantification of target acidification with pH-sensitive dye was calculated as described (49) above but the mean signal for the relevant fluorescent channel contained within the macrophage binary layer was utilized in place of the void counts. The relative indices were calculated by comparing the maximum delta from time of re-challenge between each condition and the control condition (e.g. unfed macrophages) and re-normalizing these values across biological replicates.

### 2.11 Flow cytometry

Cells were washed with FACS buffer (1x DPBS containing 0.05% BSA and 0.05% NaN_3_), incubated in Fc block (clone 2.4G2, Tonbo Biosciences) for 15 min at 4°C, and stained with the indicated antibodies for 30 min at 4°C in the dark. Cells were analyzed on an Attune Nxt flow cytometer (ThermoFisher Scientific). Background fluorescence of macrophages was determined using unstained cells rather than fluorescently-conjugated isotype controls based on previous reports (50). Flow cytometry data were analyzed using FlowJo v10.

### 2.12 Statistical analyses

Statistical analyses were performed in GraphPad Prism 9 (GraphPad Software). The statistical significance of differences in mean values was calculated using either unpaired, two-tailed Student’s t-test or one-way ANOVA where indicated. P-values less than 0.05 were considered statistically significant.

## 3 Results

### 3.1 FcγR- and complement-mediated ADCP are functionally independent, additive pathways

**Figure 1:**
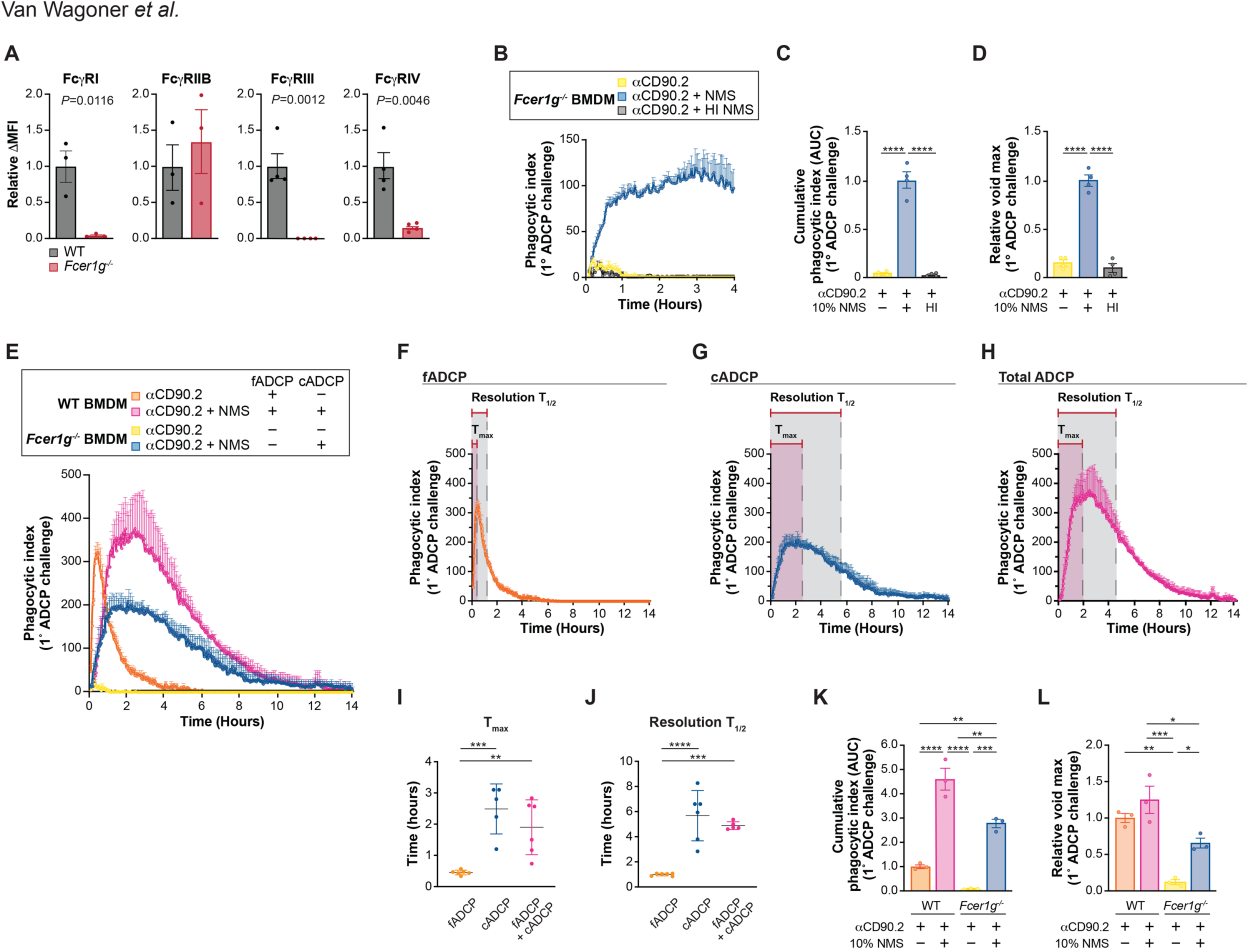
Defining fADCP and cADCP kinetics and capacity in vitro. **(A)** Validation of Fcer1g knockout bone marrow derived macrophages (BMDM) by flow cytometry. Delta MFI values for FcγRI, FcγRIIB, FcγRIII, and FcγRIV. Data shown are mean ± SEM for 3-4 independent experiments. P values were calculated using unpaired two-tailed Student’s t-tests. **(B-D)** Fcer1g-/- BMDMs were co-cultured with thymocytes at a 10:1 target:effector (T:E) ratio in the presence of 10µg/mL aCD90.2 under the following conditions: no normal mouse serum (NMS) (yellow), 10% NMS (blue), or 10% heat-inactivated (HI) NMS (black). Co-cultures were imaged for 6 minutes prior to aCD90.2 addition and imaged for another 4 hours. **(B)** Primary challenge kinetics over 4 hours, shown as a phagocytic index (mean ± SEM). **(C)** Cumulative phagocytic index (area under the curve, AUC) and **(D)** phagocytic maximum (relative void max), normalized to cADCP condition (blue). Data were analyzed by one-way ANOVA with Tukey’s multiple comparisons corrections [(C) and (D)] for four independent experiments. **(E-L)** WT or Fcer1g-/- mouse BMDMs co-cultured with thymocytes at a 10:1 T:E in the presence (pink, blue) or absence (orange, yellow) of 10% NMS. (E) Phagocytic index over 14 hours (mean ± SEM). **(F-H)** ADCP kinetics in **(E)** separated into **(F)** fADCP, **(G)** cADCP, and (H) fADCP+cADCP. Time to phagocytic maximum (Tmax) and post-maximum half-life (Resolution T1/2) are indicated. **(I-J)** Quantification of **(I)** Tmax and **(J)** Resolution T1/2 across all ADCP conditions. **(K)** AUC and **(L)** relative void max, normalized to WT fADCP condition (orange). Data were analyzed by one-way ANOVA with Tukey’s multiple comparisons corrections **(I-L)** for three independent experiments. *P < .05, **P < .01, ***P < .001, ****P < .0001. The error bars represent the mean ± SEM.

To evaluate the relative contributions of fADCP and cADCP in mAb-mediated phagocytosis and their potential interplay, we utilized an experimental live-cell imaging system that enabled us to control and measure the activation of these pathways, separately and together, in primary mouse bone marrow-derived macrophages (BMDM) co-cultured with thymocytes (49). We chose to study ADCP induced by anti-mouse CD90.2 mAb (IgG2b, clone 30-H12) because IgG2b is a strong activator of complement (51), CD90.2 is one of the most abundant surface proteins on mouse thymocytes (52, 53), and we have previously shown this clone induces robust fADCP in mouse BMDM and peritoneal macrophage (3, 53). To assess total ADCP activity (fADCP + cADCP), WT BMDM were co-cultured with anti-CD90.2-opsonized thymocytes in the presence or absence of 10% normal mouse serum (NMS) containing intact complement. To study fADCP only, the same co-culture conditions were used except that heat-inactivated (HI) NMS was used to abrogate complement activity. To study cADCP independently, we utilized BMDM from *Fcer1g^-/-^* mice, which lack the Fc receptor common gamma chain (γc) essential for surface expression and signaling of activating FcγRs (FcγRI, FcγRIII, FcγRIV), as originally reported by Takai et al. (54). Flow cytometry was used to validate this knockout, revealing significant reductions in FcγRI, FcγRIII, and FcγRIV expression in *Fcer1g^-/-^* BMDM compared to WT, with no changes in the inhibitory FcγRIIb, which functions independently of γc via an ITIM motif (**Figure 1**; **Supplementary Figure 1A**) (55–57). Accordingly, we found that *Fcer1g^-/-^* BMDM cannot carry out fADCP but do retain the capacity for cADCP (**Figures 1A–D**). We then confirmed that use of C5-depleted serum yielded ADCP results comparable to complete NMS, indicating the absence of ADCC or MAC-mediated lysis in our co-culture conditions (**Supplementary Figures 1B–D**).

In the absence of active complement, WT BMDM exhibited rapid fADCP upon addition of anti-CD90.2, with phagocytic indexes peaking at approximately 30 minutes (time to maximum, T_max_ = 0.45 hr ± 0.02 S.D.) and resolving by 2 hours (resolution T_½_ = 0.98 hr ± 0.07 S.D.) (**Figures 1E–J**; **Supplementary Figure 2A**). In contrast, *Fcer1g^-/-^* BMDM with NMS showed cADCP with a slower time to maximum (T_max_ = 1.90 hr ± 0.88 S.D.) and prolonged resolution time (resolution T_½_ = 4.88 hr ± 0.31 S.D.) (**Figures 1E–J**; **Supplementary Figure 2B**). When both pathways were active (WT BMDM + NMS), the kinetics aligned closely with cADCP, with T_max_ and resolution T_1/2_ not significantly different from cADCP alone (**Figures 1E–J**; **Supplementary Figure 2C**). The cumulative phagocytic index (AUC) was 2.75 times greater for cADCP than fADCP (**Figure 1K**), while the cumulative cell clearance achieved when both pathways were activated exceeded the individual pathways (**Figure 1K**). Maximum phagocytic activity (relative void max) was comparable between fADCP and fADCP+cADCP, with the latter being significantly greater than cADCP alone (**Figure 1L**). Taken together, these results show that fADCP and cADCP display distinct kinetic profiles and that these pathways appear to operate independently such that when activated concomitantly there is an additive effect on total cell clearance.

### 3.2 cADCP circumvents FcyR-dependent phagocytic exhaustion

**Figure 2:**
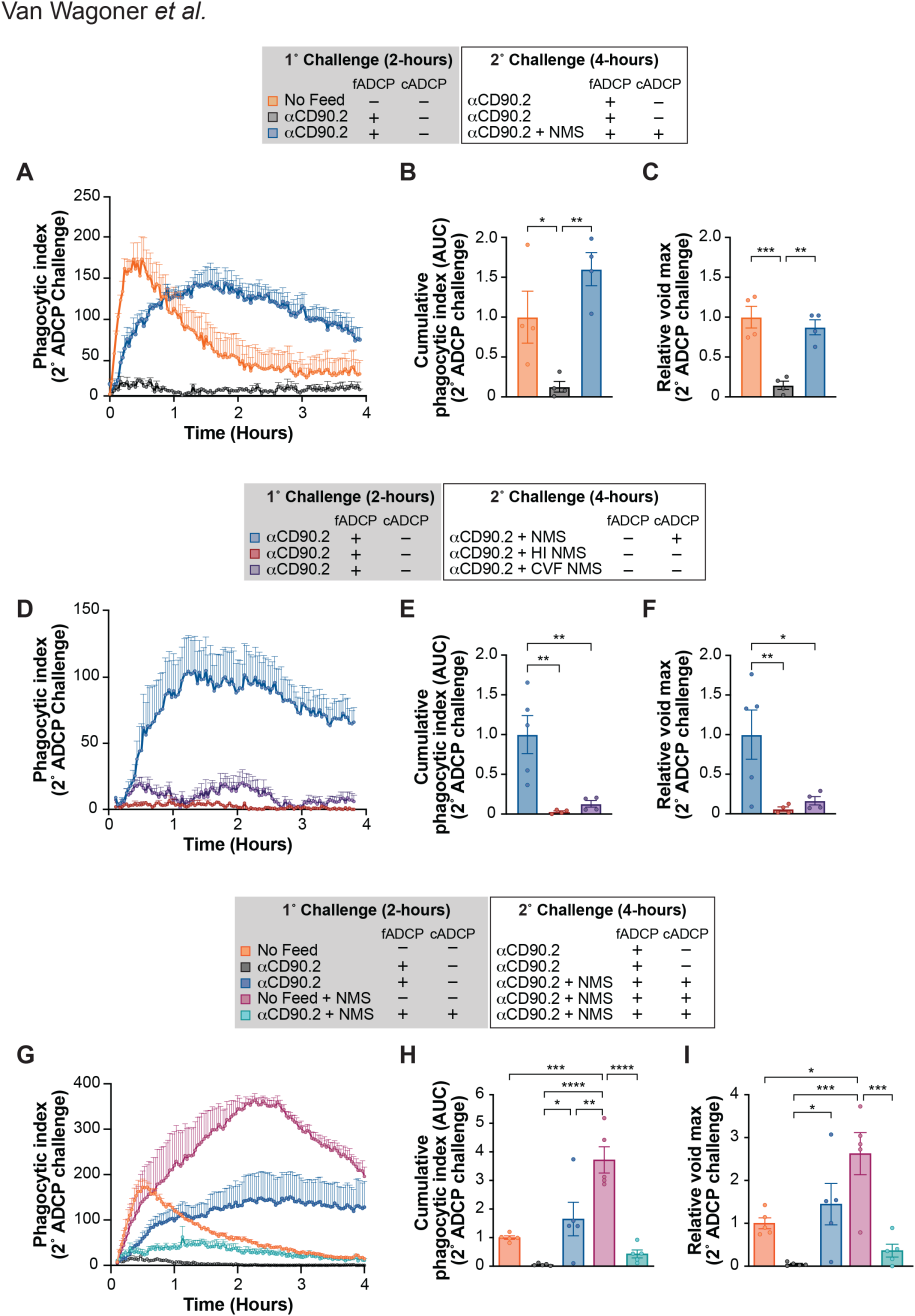
cADCP restores phagocytosis after FcγR hypophagia but exhibits its own finite capacity. **(A-C)** WT BMDM were co-cultured with thymocytes at a 10:1 T:E ratio in the absence (orange) or presence (blue, black) of 10µg/mL αCD90.2 for 2 hours. Cells were washed to remove free thymocytes and mAb, then rechallenged with fresh thymocytes (10:1 T:E) and 10µg/mL αCD90.2 for 4 hours in the presence (blue) or absence (orange, black) of NMS. **(A)** Rechallenge kinetics over 4 hours, shown as a phagocytic index (mean ± SEM). **(B)** Cumulative phagocytic index (AUC) and **(C)** relative void max for the rechallenge are quantified, normalized to the fADCP control (orange). Data were analyzed by one-way ANOVA with Tukey’s multiple comparisons corrections [(B) and (C)] for four independent experiments. **(D-F)** WT BMDM co-cultured with thymocytes (10:1 T:E) and 10µg/mL αCD90.2 for 2 hours, washed, and rechallenged as above in the presence of NMS (blue), HI NMS (crimson), or cobra venom factor (CVF) treated NMS (purple). **(D)** Rechallenge kinetics over 4 hours (mean ± SEM). **(E)** AUC and **(F)** relative void max for the rechallenge are quantified, normalized to NMS control (blue). Data were analyzed by one-way ANOVA with Tukey’s multiple comparisons corrections [(E) and (F)] for five independent experiments. **(G-I)** WT BMDM co-cultured with thymocytes (10:1 T:E) ± 10µg/mL αCD90.2 and ± 10% NMS for 2 hours, washed, and rechallenged with fresh thymocytes (10:1 T:E) and 10µg/mL αCD90.2 ± 10% NMS for 4 hours. **(G)** Rechallenge kinetics over 4 hours (mean ± SEM). **(H)** AUC and **(I)** relative void max for the rechallenge are quantified, normalized to the unfed controls rechallenged under fADCP conditions (blue). Data were analyzed by one-way ANOVA with Tukey’s multiple comparisons corrections [(H) and (I)] for five independent experiments. *P < .05, **P < .01, ***P < .001, ****P <.0001. The error bars represent the mean ± SEM.

In a recent study of fADCP using WT macrophages, we demonstrated that after an initial encounter with antibody-opsonized targets, macrophages exhibit a near-complete loss of FcγR-dependent phagocytic activity upon rechallenge with fresh targets and antibody (3). This state of phagocytic exhaustion, which we termed “hypophagia,” persists for 24–48 hours and appears to be specific for fADCP, as hypophagic macrophages show no defect in apoptotic cell phagocytosis (efferocytosis) (3). To determine whether cADCP could provide additional phagocytic capacity in macrophages undergoing fADCP exhaustion, we performed rechallenge experiments in WT BMDM in the presence or absence of complement. Macrophages were initially challenged with anti-CD90.2-opsonized thymocytes in the absence of NMS to induce fADCP, washed to remove unbound targets and antibody and rechallenged with fresh opsonized thymocytes in the presence or absence of NMS. Consistent with our prior fADCP hypophagia study, rechallenge in the absence of complement resulted in minimal additional phagocytosis due to exhaustion of the fADCP pathway (**Figures 2A–C**). However, addition of NMS prior to rechallenge restored robust phagocytosis, with capacity comparable to unfed controls rechallenged under fADCP conditions (**Figures 2A–C**), indicating that cADCP is fully functional when the fADCP pathway is exhausted. To verify complement dependence, we repeated the experiment using heat-inactivated or cobra venom factor (CVF)-treated NMS. Heat inactivation is commonly used to inactivate the complement pathway via denaturation of its components, while CVF, a complement activating C3 analog, depletes complement by causing the consumption of its components (58–61). Strikingly, both treatments caused a near-complete loss of phagocytosis upon rechallenge (**Figures 2D–F**), confirming that cADCP functions normally even when the fADCP pathway is exhausted, further substantiating the functional independence of these pathways.

To assess whether cADCP, like fADCP, has a finite capacity leading to hypophagia, we performed analogous rechallenge experiments with NMS included in primary and secondary challenges with WT BMDM. To enable a direct comparison of fADCP and cADCP phagocytic capacity, we also included the conditions in **Figure 2A** in these experiments. Macrophages initially challenged under fADCP + cADCP (teal) conditions showed markedly reduced activity upon rechallenge compared to unfed control macrophages rechallenged under identical conditions (**Figures 2G–I**, maroon). Compared to fADCP hypophagia in the same experiment (orange vs. black) where loss of phagocytic activity is >90%, total ADCP activity (fADCP + cADCP) was similarly reduced by prior feeding with fADCP + cADCP conditions (**Figures 2G, H**, maroon vs. teal). Similarly, peak void maximum of a fADCP + cADCP rechallenge was significantly reduced in macrophages that were previously challenged under fADCP + cADCP conditions (**Figures 2G–I**, maroon vs. teal). Because we found that macrophages in state of fADCP hypophagia can still engulf mAb-opsonized targets via cADCP, these results suggest that, like fADCP, the cADCP pathway is finite and exhaustible.

### 3.3 cADCP capacity as a function of target load

**Figure 3:**
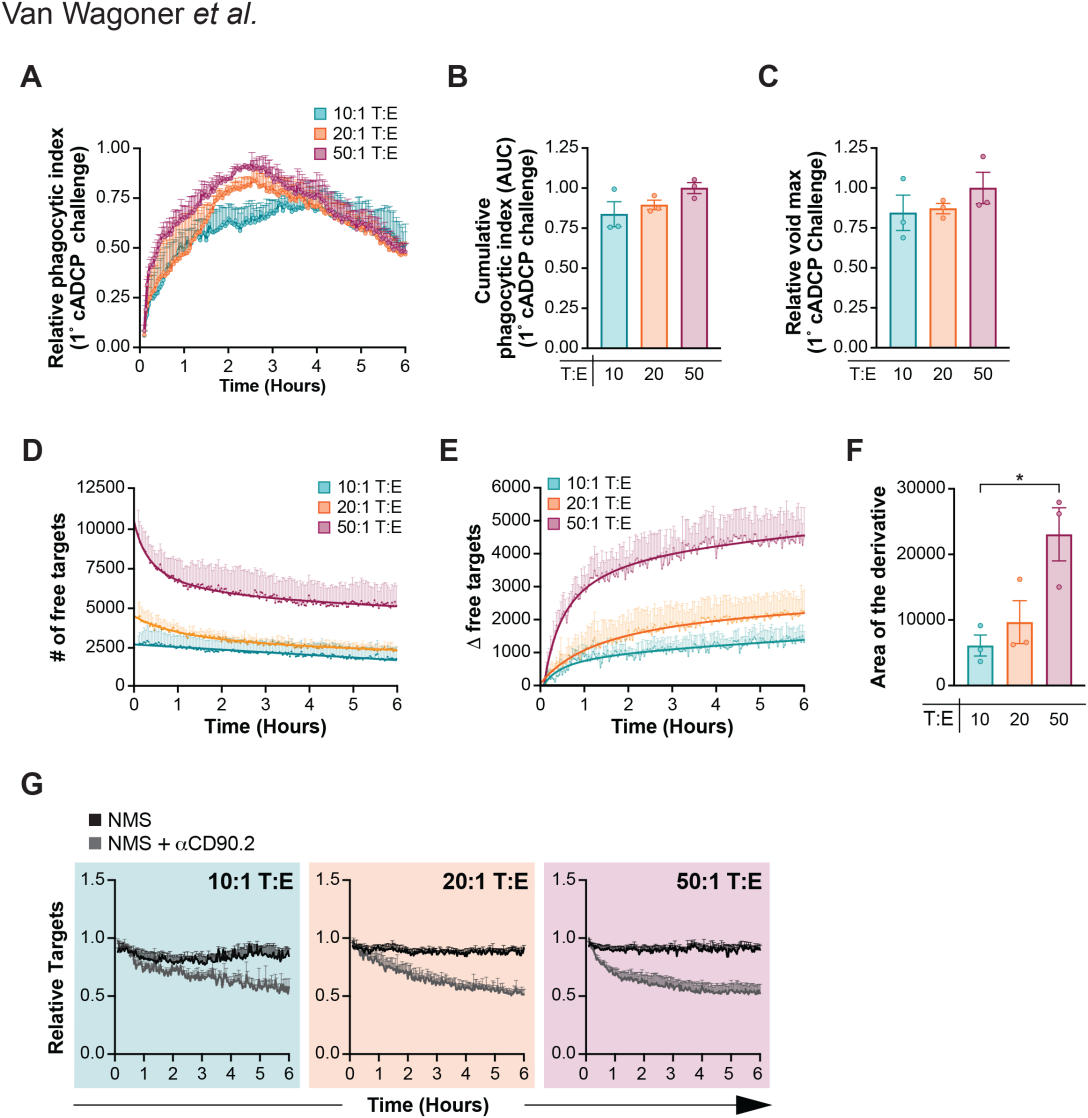
cADCP capacity as a function of target load. **(A-G)***Fcer1g^-/-^* BMDM were co-cultured with thymocytes at 10:1 (teal), 20:1 (orange), or 50:1 (maroon) T:E ratios in the presence of 10µg/mL αCD90.2 and 10% NMS for 6 hours. **(A)** Primary challenge kinetics over 6 hours, shown as a relative phagocytic index (mean ± SEM), normalized to the maximum void count across all conditions. **(B)** Cumulative phagocytic index (AUC) and **(C)** relative void maximum, each normalized to the 50:1 T:E cADCP condition (maroon). Data were analyzed by one-way ANOVA with Tukey’s multiple comparisons corrections [(B) and (C)] for three independent experiments and showed no significance. (D-G) Orthogonal assessment of phagocytosis by brightfield quantification of free targets. *Fcer1g^-/-^* BMDM were co-cultured as described above, with values normalized to each condition’s void maximum. (D) Number of free targets over 6 hours for each T:E ratio. **(E)** Change in free target number relative to the initial frame. **(F)** Area of the first derivative curve, analyzed by one-way ANOVA with Tukey’s multiple comparisons test for three independent experiments. Data shown are mean ± SEM; *P < .05. **(G)** Free target counts under cADCP conditions normalized to no-antibody controls, confirming that target reduction was not due to cells shifting out of the imaging field.

Having found that cADCP phagocytic capacity is nearly three times greater than fADCP (**Figures 1E–K**), we further explored cADCP phagocytic capacity in isolation using *Fcer1g^-/-^* BMDM and variable target:effector (T:E ratio) at 10, 20, and 50 for six hours. Surprisingly, we found that varying the T:E did not significantly affect the magnitude or cumulative outcome of cADCP as measured by the microscopic void detection approach (**Figures 3A–C**). Indeed, we found that at 20 and 50 T:E ratios, individual phagocytic voids became harder to resolve by microscopy (**Supplementary Figure 3**). Therefore, we used an orthogonal approach to our void-based method and measured cADCP activity by quantifying the number of free (unengulfed) targets as a proxy to engulfment (**Figure 3A**). Free target numbers declined over time in the co-cultures in an T:E-dependent manner (**Figure 3D**), with the change in free targets (Δ) yielding a larger derivative area at 50:1 compared to 10:1 (**Figures 3E, F**). We confirmed that the Δ free target values in **Figures 3E, F** reflected antibody-specific clearance by comparing target cell numbers in each frame over time with and without anti-CD90.2 (**Figure 3G**). Finally, complement-dependent cytotoxicity (CDC) cannot explain the decline in target cell numbers in the co-cultures over time as no difference was seen in cADCP with or without C5, a key protein involved in the lytic, terminal phase of complement activation (62, 63) (**Supplementary Figures 1B, C**). Collectively, these data show that cADCP capacity upon initial challenge is dependent on target cell availability (**Supplementary Figure 2**), further reinforcing the greater capacity of cADCP compared to fADCP.

### 3.4 Phagocytic capacity of cADCP pathway

**Figure 4:**
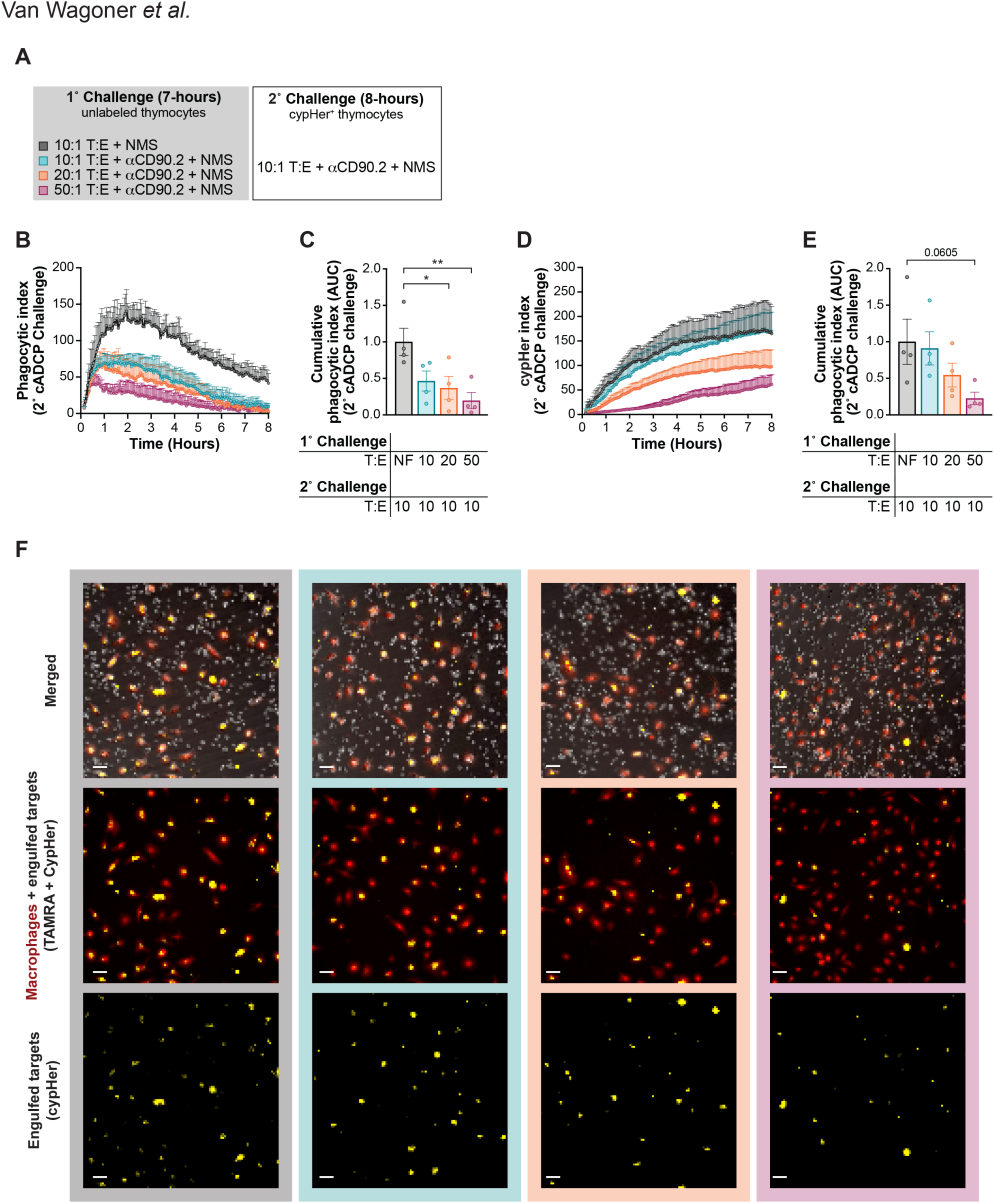
Phagocytic capacity of cADCP pathway. **(A)** Experimental set up for B-F. Fcer1g-/-BMDM were co-cultured with unlabeled thymocytes at 10:1 (teal), 20:1 (orange), or 50:1 (maroon) T:E ratios in the presence of 10µg/mL αCD90.2 and 10% NMS for 7 hours. Control *Fcer1g^-/-^* BMDM were co-cultured with thymocytes at a 10:1 T:E in 10% NMS without opsonizing mAb (black). BMDM were washed and rechallenged with cypHer5E-labeled target thymocytes at a 10:1 T:E, in the presence of 10µg/mL αCD90.2 and 10% NMS, for an additional 8 hours. **(B)** Void based detection of target cell clearance during the 8-hour rechallenge, shown as a phagocytic index (mean ± SEM). **(C)** Cumulative phagocytic index (AUC), normalized to previously non-fed (NF) *Fcer1g^-/-^* BMDM controls (black). **(D)** Dye-based detection of phagocytosis using live-cell imaging of *Fcer1g^-/-^* BMDM co-cultured with cypHer5E-labeled thymocytes (a pH-sensitive dye that increases fluorescence upon internalization and acidification in the phagolysosomal pathway). CypHer index is shown over the 8-hour rechallenge. **(E)** AUC quantified from (D), normalized to previously NF controls (black). **(F)** Representative images from the final timepoint of the rechallenge showing cypHer5E signal in *Fcer1g^-/-^* BMDM labeled with cell-permeant fluorescent tracer dye TAMRA. Data were analyzed by one-way ANOVA corrected with Dunnett’s multiple comparisons to the control mean [(C) and (E)] for four independent experiments. Data shown are mean ± SEM; *P < .05, **P < .01.

We next tested how varying the target cell load affected the overall phagocytic capacity of the cADCP pathway. For this, we challenged *Fcer1g^-/-^* BMDM with anti-CD90.2 and unlabeled thymocytes at varying T:E ratios (or no-feed controls) for 7 hours, washed, and then rechallenged with fresh mAb and 10:1 thymocytes labeled with the pH-sensitive dye cypHer5E (**Figure 4A**) (64). Void-based phagocytic indexes (AUC) during rechallenge decreased inversely with primary challenge T:E, reflecting dose-dependent exhaustion (**Figures 4B, C**). Normalized AUC was significantly reduced under 20:1 and 50:1 T:E primary challenge conditions relative to no-feed controls (**Figure 4C**). CypHer5E indexes independently validated this, showing diminished signal with increasing primary target cell load (**Figure 4D**), with corresponding AUC reductions (**Figure 4E**). Representative merged images at rechallenge endpoints illustrate fewer CypHer-positive macrophages after higher primary T:E challenges (**Figure 4F**). These concordant void- and dye-based findings confirm that cADCP hypophagia is profound and scales with engulfment burden.

### 3.5 cADCP hypophagia is transient

**Figure 5:**
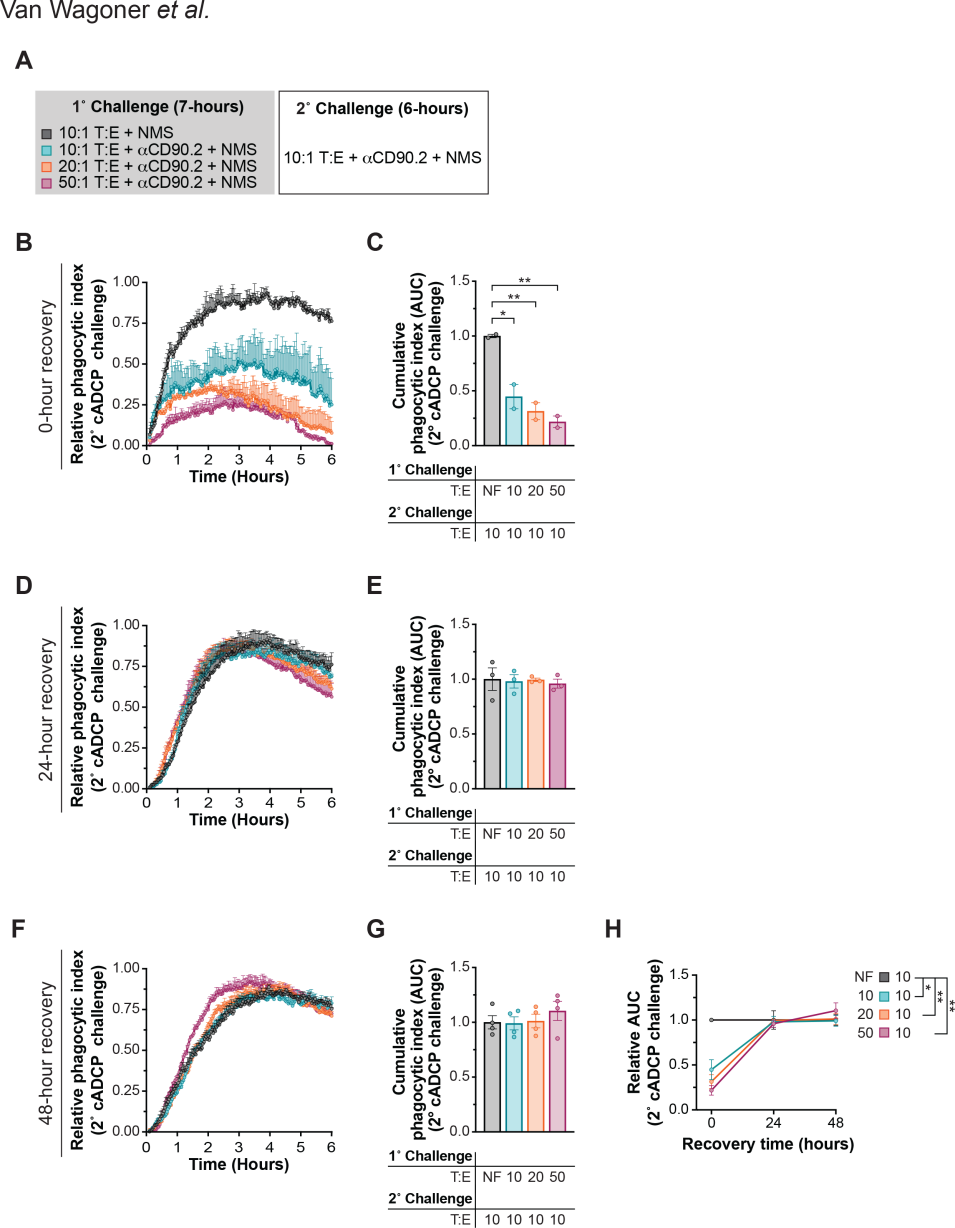
cADCP hypophagia is transient. **(A)** Experimental set up for B-H. *Fcer1g^-/-^* BMDM were co-cultured with thymocytes at 10:1 (teal), 20:1 (orange), or 50:1 (maroon) T:E ratios in the presence of 10µg/mL αCD90.2 and 10% NMS for 7 hours. Control Fcer1g-/- BMDM were co-cultured with thymocytes at a 10:1 T:E ratio in 10% NMS without opsonizing mAb (black). BMDM were washed and allowed to recover for 0-, 24, or 48-hours before rechallenging with thymocytes (10:1 T:E), 10µg/mL αCD90.2, and 10% NMS for 6 hours. **(B, D, F)** Rechallenge kinetics over 6 hours, following a 0-, 24-, or 48-hour recovery, respectively. **(C, E, G)** Cumulative phagocytic index (AUC) of each rechallenge condition after a 0, 24, or 48-hour recovery, respectively, normalized to previously non-fed (NF) *Fcer1g^-/-^* BMDM controls (black). (H) Summary of relative AUC across recovery time points. Data were analyzed by one-way ANOVA corrected with Dunnett’s multiple comparisons test against the control mean [(C) and (E) and (G)] for four independent experiments. Data shown are mean ± SEM; *P < .05, **P < .01.

To determine the persistence of cADCP hypophagia, we allowed *Fcer1g^-/-^* BMDM to recover for 0–48 hours following a primary challenge with anti-CD90.2-opsonized thymocytes at T:E’s of 10, 20, or 50 (**Figure 5A**). cADCP activity was then measured anti-CD90.2-opsonized thymocytes at a 10:1 T:E. As shown in **Figure 4**, with no recovery time, rechallenge phagocytic indexes were significantly reduced for all T:E (**Figures 5B, C**). At 12, 24 and 48 hours, indexes approached no-feed levels across all T:E, with no significant AUC differences observed (**Figures 5D–G**; **Supplementary Figure 4**). A summary plot of relative AUC over recovery time underscores the brief and reversible nature of cADCP hypophagia (**Figure 5H**), distinct from the more prolonged recovery observed in fADCP (3). These kinetics highlight effector exhaustion as a shared but pathway-specific constraint on ADCP-mediated cytotoxicity.

## 4 Discussion

Complement activation by antibodies via the classical pathway leads to opsonization of target cells with C3 derived fragments. These covalently bound peptides can ligate macrophage complement receptors resulting in phagocytosis of opsonized target cells (62, 63). Using a vital, real-time assay of phagocytosis (3, 49), we demonstrate that cADCP-mediated cytotoxicity induced by complement activating antibodies is of a greater magnitude than fADCP and exhibits distinct kinetics, as well as differences in the attenuation and resolution of phagocytic activity.

Previous studies have shown that antibody-mediated complement activation results in the deposition of complement fragments on target cells and subsequent induction of cADCP (23, 26, 32, 33, 38, 65, 66). In this study, using a quantitative kinetic method, we show for the first time that mAb-induced cADCP and fADCP have distinct kinetic profiles and overall target cell clearance capacities. By precisely controlling the co-culture conditions of murine macrophages and target thymocytes opsonized with anti-CD90.2, we quantified phagocytosis selectively induced by cADCP (*Fcer1g^-/-^* BMDM and NMS) or fADCP (WT BMDM without NMS).

fADCP was characterized by a brief period of rapid phagocytosis followed by attenuation (hypophagia), resulting in significantly less cumulative phagocytosis than cADCP. In contrast, cADCP exhibited a slower rise in phagocytic index and a lower relative void index yet achieved greater overall target cell clearance. Concomitant engagement of fADCP and cADCP produced a significantly increased cumulative phagocytic index compared to either mechanism alone, indicating additive phagocytic capacity. Importantly, cADCP is also finite: over time, the phagocytic index declined, and macrophage engulfment was reduced upon rechallenge. The depth of this attenuation significantly increased with higher initial effector-to-target (T:E) ratios, although cADCP activity recovered within 24 hours across all T:E ratios tested. Together, these data indicate that ADCP can be induced by FcγR, complement receptors, or both simultaneously, with what appear to be additive effects on phagocytic capacity. The distinct kinetics and attenuation profiles of cADCP relative to fADCP highlight mechanistic differences that require additional study. These findings enhance our understanding of the physiological and pathological activities of Ab and inform strategies to optimize their therapeutic efficacy.

Clearance of Ab opsonized cells by macrophages is a fundamental physiological mechanism that contributes to tissue homeostasis, including red blood cell turnover, and the elimination of pathogens that induce humoral immune responses (66–72). Given that human IgM and some isotypes of IgG can efficiently activate complement (37, 40, 73–77), our data on cADCP could be useful in better understanding these mechanisms. Beyond homeostatic processes, pathological complement activation is also implicated in autoimmune diseases (e.g. autoimmune hemolytic anemia) (78) and infection induced diseases (e.g. microangiopathic hemolytic anemia and COVID-19) (79, 80). By characterizing the kinetics and durability of cADCP, our study offers a more detailed understanding of how complement-dependent phagocytosis may operate in both normal and disease associated contexts. Our findings warrant consideration in future therapeutic investigations, particularly when evaluating strategies to modulate complement for clinical benefit.

It is important to acknowledge the limitations of the present work. While Pinney et al. demonstrated that murine BMDMs provide a reasonable representation of fADCP across multiple models *in vivo* and *in vitro*, the distinct kinetics and regulation of cADCP observed here indicate that the same translational fidelity cannot be assumed for complement-dependent mechanisms. Consequently, *in vivo* validation and confirmation of these findings in human macrophages represent important next steps.

mAbs have an increasingly important role in the management of diseases caused by immune cell dysregulation or neoplasia. The development of the unconjugated anti-CD20 mAb rituximab marked a paradigm shift in the treatment of B-cell mediated pathology, especially lymphoma. Although the mechanisms underlying mAb efficacy were initially poorly defined, it is now well established that mAb induced ADCP represents a major cytotoxic mechanism (1–11). Our data further indicate that this critical process may substantially rely on cADCP, highlighting an underappreciated role for complement activation in mAb therapy. Early efforts to enhance fADCP focused on mAb Fc engineering strategies that reduced complement activation (25, 27, 30, 34, 39, 81, 82), which could inadvertently constrain mAb efficacy by diminishing cADCP contributions. In contrast, more recent approaches that engineer mAb Fc domains to promote hexamerization to augment complement activation (35–37, 83) may enhance cADCP and improve treatment efficacy. Together with our findings, these data indicate that fADCP and cADCP act as complementary, non-overlapping contributors to innate immune cytotoxicity, suggesting that rational modulation of complement engagement represents a promising strategy for optimizing antibody-based therapies.

## Data Availability

The raw data supporting the conclusions of this article will be made available by the authors, without undue reservation.

## References

[1] Church AK VanDerMeid KR Baig NA Baran AM Witzig TE Nowakowski GS . Anti-CD20 monoclonal antibody-dependent phagocytosis of chronic lymphocytic leukemia cells by autologous macrophages. Clin Exp Immunol. (2016) 183:90–101. doi: 10.1111/cei.12697. PMID: 26307241 PMC4687519

[2] Zent CS Elliott MR . Maxed out macs: physiologic cell clearance as a function of macrophage phagocytic capacity. FEBS J. (2017) 284:1021–39. doi: 10.1111/febs.13961. PMID: 27863012 PMC5378628

[3] Pinney JJ Rivera-Escalera F Chu CC Whitehead HE VanDerMeid KR Nelson AM . Macrophage hypophagia as a mechanism of innate immune exhaustion in mAb-induced cell clearance. Blood. (2020) 136:2065–79. doi: 10.1182/blood.2020005571. PMID: 32556153 PMC7596847

[4] Van Wagoner CM Rivera-Escalera F Jaimes-Delgadillo NC Chu CC Zent CS Elliott MR . Antibody-mediated phagocytosis in cancer immunotherapy. Immunol Rev. (2023) 319:128–41. doi: 10.1111/imr.13265. PMID: 37602915 PMC10615698

[5] Lefebvre M-L Krause SW Salcedo M Nardin A . Ex vivo-activated human macrophages kill chronic lymphocytic leukemia cells in the presence of rituximab: mechanism of antibody-dependent cellular cytotoxicity and impact of human serum. J Immunother. (2006) 29:388–97. doi: 10.1097/01.cji.0000203081.43235.d7. PMID: 16799334

[6] VanDerMeid KR Elliott MR Baran AM Barr PM Chu CC Zent CS . Cellular cytotoxicity of next-generation CD20 monoclonal antibodies. Cancer Immunol Res. (2018) 6:1150–60. doi: 10.1158/2326-6066.CIR-18-0319. PMID: 30089638

[7] Gül N Babes L Siegmund K Korthouwer R Bögels M Braster R . Macrophages eliminate circulating tumor cells after monoclonal antibody therapy. J Clin Invest. (2014) 124:812–23. doi: 10.1172/JCI66776. PMID: 24430180 PMC3904600

[8] Taylor RP Lindorfer MA . Cytotoxic mechanisms of immunotherapy: Harnessing complement in the action of anti-tumor monoclonal antibodies. Semin Immunol. (2016) 28:309–16. doi: 10.1016/j.smim.2016.03.003. PMID: 27009480

[9] Montalvao F Garcia Z Celli S Breart B Deguine J Van Rooijen N . The mechanism of anti-CD20-mediated B cell depletion revealed by intravital imaging. J Clin Invest. (2013) 123:5098–103. doi: 10.1172/JCI70972. PMID: 24177426 PMC3859399

[10] Grandjean CL Montalvao F Celli S Michonneau D Breart B Garcia Z . Intravital imaging reveals improved Kupffer cell-mediated phagocytosis as a mode of action of glycoengineered anti-CD20 antibodies. Sci Rep. (2016) 6:34382. doi: 10.1038/srep34382. PMID: 27698437 PMC5048169

[11] Weiskopf K Weissman IL . Macrophages are critical effectors of antibody therapies for cancer. MAbs. (2015) 7:303–10. doi: 10.1080/19420862.2015.1011450. PMID: 25667985 PMC4622600

[12] Flannagan RS Jaumouillé V Grinstein S . The cell biology of phagocytosis. Annu Rev Pathology: Mech Dis. (2012) 7:61–98. doi: 10.1146/annurev-pathol-011811-132445. PMID: 21910624

[13] Battella S Cox MC Santoni A Palmieri G . Natural killer (NK) cells and anti-tumor therapeutic mAb: unexplored interactions. J Leukocyte Biol. (2016) 99:87–96. doi: 10.1189/jlb.5vmr0415-141r. PMID: 26136506

[14] Van Der Bij GJ Bögels M Otten MA Oosterling SJ Kuppen PJ Meijer S . Experimentally induced liver metastases from colorectal cancer can be prevented by mononuclear phagocyte-mediated monoclonal antibody therapy. J Hepatol. (2010) 53:677–85. doi: 10.1016/j.jhep.2010.04.023. PMID: 20619916

[15] Oflazoglu E Stone IJ Brown L Gordon KA van Rooijen N Jonas M . Macrophages and Fc-receptor interactions contribute to the antitumor activities of the anti-CD40 antibody SGN-40. Br J Cancer. (2009) 100:113–7. doi: 10.1038/sj.bjc.6604812. PMID: 19066610 PMC2634668

[16] Law C-L Gordon KA Collier J Klussman K McEarchern JA Cerveny CG . Preclinical antilymphoma activity of a humanized anti-CD40 monoclonal antibody, SGN-40. Cancer Res. (2005) 65:8331–46. doi: 10.1158/0008-5472.CAN-05-0095. PMID: 16166310

[17] Minard-Colin V Xiu Y Poe JC Horikawa M Magro CM Hamaguchi Y . Lymphoma depletion during CD20 immunotherapy in mice is mediated by macrophage FcγRI, FcγRIII, and FcγRIV. Blood. (2008) 112:1205–13. doi: 10.1182/blood-2008-01-135160. PMID: 18495955 PMC2515149

[18] Gong Q Ou Q Ye S Lee WP Cornelius J Diehl L . Importance of cellular microenvironment and circulatory dynamics in B cell immunotherapy1. J Immunol. (2005) 174:817–26. doi: 10.4049/jimmunol.174.2.817. PMID: 15634903

[19] Golay J Cittera E Di Gaetano N Manganini M Mosca M Nebuloni M . The role of complement in the therapeutic activity of rituximab in a murine B lymphoma model homing in lymph nodes. Hematologica. (2006) 91:176–83. 16461301

[20] Beum PV Lindorfer MA Taylor RP . Within peripheral blood mononuclear cells, antibody-dependent cellular cytotoxicity of rituximab-opsonized Daudi cells is promoted by NK cells and inhibited by monocytes due to shaving. J Immunol. (2008) 181:2916–24. doi: 10.4049/jimmunol.181.4.2916. PMID: 18684983

[21] Uchiyama S Suzuki Y Otake K Yokoyama M Ohta M Aikawa S . Development of novel humanized anti‐CD20 antibodies based on affinity constant and epitope. Cancer Sci. (2010) 101:201–9. doi: 10.1111/j.1349-7006.2009.01392.x. PMID: 19930155 PMC11158754

[22] Beum PV Lindorfer MA Beurskens F Stukenberg PT Lokhorst HM Pawluczkowycz AW . Complement activation on B lymphocytes opsonized with rituximab or ofatumumab produces substantial changes in membrane structure preceding cell lysis. J Immunol. (2008) 181:822–32. doi: 10.4049/jimmunol.181.1.822. PMID: 18566448

[23] Teeling JL . Characterization of new human CD20 monoclonal antibodies with potent cytolytic activity against non-Hodgkin lymphomas. Blood. (2004) 104:1793–800. doi: 10.1182/blood-2004-01-0039. PMID: 15172969

[24] Teeling JL Mackus WJM Wiegman LJJM Van Den Brakel JHN Beers SA French RR . The biological activity of human CD20 monoclonal antibodies is linked to unique epitopes on CD20. J Immunol. (2006) 177:362–71. doi: 10.4049/jimmunol.177.1.362. PMID: 16785532

[25] Klein C Lammens A Schäfer W Georges G Schwaiger M Mössner E . Epitope interactions of monoclonal antibodies targeting CD20 and their relationship to functional properties. mAbs. (2013) 5:22–33. doi: 10.4161/mabs.22771. PMID: 23211638 PMC3564883

[26] Zent CS Pinney JJ Chu CC Elliott MR . Complement activation in the treatment of B-cell Malignancies. Antibodies. (2020) 9:68. doi: 10.3390/antib9040068. PMID: 33271825 PMC7709106

[27] Mössner E Brünker P Moser S Püntener U Schmidt C Herter S . Increasing the efficacy of CD20 antibody therapy through the engineering of a new type II anti-CD20 antibody with enhanced direct and immune effector cell–mediated B-cell cytotoxicity. Blood. (2010) 115:4393–402. doi: 10.1182/blood-2009-06-225979. PMID: 20194898 PMC2881503

[28] Tobinai K Ogura M Kobayashi Y Uchida T Watanabe T Oyama T . Phase I study of LY2469298, an Fc-engineered humanized anti-CD20 antibody, in patients with relapsed or refractory follicular lymphoma. Cancer Sci. (2011) 102:432–8. doi: 10.1111/j.1349-7006.2010.01809.x. PMID: 21205069

[29] Liu R Oldham R Teal E Beers S Cragg M . Fc-engineering for modulated effector functions—improving antibodies for cancer treatment. Antibodies. (2020) 9:64. doi: 10.3390/antib9040064. PMID: 33212886 PMC7709126

[30] Beers SA Chan CHT James S French RR Attfield KE Brennan CM . Type II (tositumomab) anti-CD20 monoclonal antibody out performs type I (rituximab-like) reagents in B-cell depletion regardless of complement activation. Blood. (2008) 112:4170–7. doi: 10.1182/blood-2008-04-149161. PMID: 18583569 PMC2582008

[31] Uchida J Hamaguchi Y Oliver JA Ravetch JV Poe JC Haas KM . The innate mononuclear phagocyte network depletes B lymphocytes through Fc receptor–dependent mechanisms during anti-CD20 antibody immunotherapy. J Exp Med. (2004) 199:1659–69. doi: 10.1084/jem.20040119. PMID: 15210744 PMC2212805

[32] Golay J Taylor RP . The role of complement in the mechanism of action of therapeutic anti-cancer mAbs. Antibodies (Basel). (2020) 9:58. doi: 10.3390/antib9040058. PMID: 33126570 PMC7709112

[33] Di Gaetano N Cittera E Nota R Vecchi A Grieco V Scanziani E . Complement activation determines the therapeutic activity of rituximab *in vivo*. J Immunol. (2003) 171:1581–7. doi: 10.4049/jimmunol.171.3.1581. PMID: 12874252

[34] Cragg MS Glennie MJ . Antibody specificity controls *in vivo* effector mechanisms of anti-CD20 reagents. Blood. (2004) 103:2738–43. doi: 10.1182/blood-2003-06-2031. PMID: 14551143

[35] Beurskens FJ de Jong RN Verploegen S Voorhorst M Strumane K Lindorfer MA . Enhanced IgG hexamerization mediates efficient C1q docking and complement-dependent cytotoxicity; preclinical proof of concept on primary CLL and Burkitt lymphoma. Blood. (2013) 122:375. doi: 10.1182/blood.V122.21.375.375. PMID: 41761659

[36] Abendstein L Dijkstra DJ Tjokrodirijo RTN van Veelen PA Trouw LA Hensbergen PJ . Complement is activated by elevated IgG3 hexameric platforms and deposits C4b onto distinct antibody domains. Nat Commun. (2023) 14:4027. doi: 10.1038/s41467-023-39788-5. PMID: 37419978 PMC10328927

[37] Diebolder CA Beurskens FJ de Jong RN Koning RI Strumane K Lindorfer MA . Complement is activated by IgG hexamers assembled at the cell surface. Science. (2014) 343:1260–3. doi: 10.1126/science.1248943. PMID: 24626930 PMC4250092

[38] Lee C-H Romain G Yan W Watanabe M Charab W Todorova B . IgG Fc domains that bind C1q but not effector Fcγ receptors delineate the importance of complement-mediated effector functions. Nat Immunol. (2017) 18:889–98. doi: 10.1038/ni.3770. PMID: 28604720 PMC6015732

[39] Illidge T Klein C Sehn LH Davies A Salles G Cartron G . Obinutuzumab in hematologic Malignancies: Lessons learned to date. Cancer Treat Rev. (2015) 41:784–92. doi: 10.1016/j.ctrv.2015.07.003. PMID: 26190254

[40] Oskam N Ooijevaar-de Heer P Derksen NIL Kruithof S de Taeye SW Vidarsson G . At critically low antigen densities, IgM hexamers outcompete both IgM pentamers and IgG1 for human complement deposition and complement-dependent cytotoxicity. J Immunol. (2022) 209:16–25. doi: 10.4049/jimmunol.2101196. PMID: 35705253

[41] Bond A Morrissey MA . Biochemical and biophysical mechanisms macrophages use to tune phagocytic appetite. J Cell Sci. (2025) 138:JCS263513. doi: 10.1242/jcs.263513. PMID: 39749603 PMC11828473

[42] Cannon GJ Swanson JA . The macrophage capacity for phagocytosis. J Cell Sci. (1992) 101:907–13. doi: 10.1242/jcs.101.4.907. PMID: 1527185

[43] Winer BY Settle AH Yakimov AM Jeronimo C Lazarov T Tipping M . Plasma membrane abundance dictates phagocytic capacity and functional crosstalk in myeloid cells. (2023). doi: 10.1101/2023.09.12.556572, PMID: 38848343 PMC11485225

[44] Settle AH Winer BY De Jesus MM Seeman L Wang Z Chan E . β2 integrins impose a mechanical checkpoint on macrophage phagocytosis. Nat Commun. (2024) 15:8182. doi: 10.1038/s41467-024-52453-9. PMID: 39294148 PMC11411054

[45] Bond A Fiaz S Rollins K Nario JEQ Snyder ET Atkins DJ . Prior Fc receptor activation primes macrophages for increased sensitivity to IgG via long-term and short-term mechanisms. Dev Cell. (2024) 59:2882–96.e7. doi: 10.1016/j.devcel.2024.07.017. PMID: 39137774 PMC11537821

[46] Available online at: https://docs.abcam.com/pdf/misc/abcam-protocols-book.pdf. abcam-protocols-book.pdf (Accessed October 6, 2025).

[47] Murphy PS Wang J Bhagwat SP Munger JC Janssen WJ Wright TW . CD73 regulates anti-inflammatory signaling between apoptotic cells and endotoxin-conditioned tissue macrophages. Cell Death Differ. (2017) 24:559–70. doi: 10.1038/cdd.2016.159. PMID: 28060378 PMC5344214

[48] Stevenson C De La Rosa G Anderson CS Murphy PS Capece T Kim M . Essential role of Elmo1 in Dock2-dependent lymphocyte migration. J Immunol. (2014) 192:6062–70. doi: 10.4049/jimmunol.1303348. PMID: 24821968 PMC4127066

[49] Chu CC Pinney JJ Whitehead HE Rivera-Escalera F VanDerMeid KR Zent CS . High-resolution quantification of discrete phagocytic events by live cell time-lapse high-content microscopy imaging. J Cell Sci. (2020) 133:jcs237883. doi: 10.1242/jcs.237883. PMID: 32005699 PMC7075070

[50] Keeney M Gratama JW Chin-Yee IH Sutherland DR . Isotype controls in the analysis of lymphocytes and CD34+ stem and progenitor cells by flow cytometry--time to let go! Cytometry. (1998) 34:280–3. doi: 10.1002/(sici)1097-0320(19981215)34:6<280::aid-cyto6>3.0.co;2-h 9879645

[51] Nimmerjahn F Ravetch JV . Fcγ receptors as regulators of immune responses. Nat Rev Immunol. (2008) 8:34–47. doi: 10.1038/nri2206. PMID: 18064051

[52] Shiku H Kisielow P Bean MA Takahashi T Boyse EA Oettgen HF . Expression of T-cell differentiation antigens on effector cells in cell-mediated cytotoxicity *in vitro*. Evidence for functional heterogeneity related to the surface phenotype of T cells. J Exp Med. (1975) 141:227–41. doi: 10.1084/jem.141.1.227. PMID: 1078839 PMC2190510

[53] Ledbetter JA Rouse RV Micklem HS Herzenberg LA . T cell subsets defined by expression of Lyt-1,2,3 and Thy-1 antigens. Two-parameter immunofluorescence and cytotoxicity analysis with monoclonal antibodies modifies current views. J Exp Med. (1980) 152:280–95. doi: 10.1084/jem.152.2.280. PMID: 6156984 PMC2185937

[54] Takai T Li M Sylvestre D Clynes R Ravetch JV . FcR γ chain deletion results in pleiotrophic effector cell defects. Cell. (1994) 76:519–29. doi: 10.1016/0092-8674(94)90115-5. PMID: 8313472

[55] Daëron M Latour S Malbec O Espinosa E Pina P Pasmans S . The same tyrosine-based inhibition motif, in the intra-cytoplasmic domain of FcγRIIB, regulates negatively BCR-, TCR-, and FcR-dependent cell activation. Immunity. (1995) 3:635–46. doi: 10.1016/1074-7613(95)90134-5. PMID: 7584153

[56] Ravetch JV Lanier LL . Immune inhibitory receptors. Science. (2000) 290:84–9. doi: 10.1126/science.290.5489.84. PMID: 11021804

[57] Clynes RA Towers TL Presta LG Ravetch JV . Inhibitory Fc receptors modulate *in vivo* cytoxicity against tumor targets. Nat Med. (2000) 6:443–6. doi: 10.1038/74704. PMID: 10742152

[58] Soltis RD Hasz D Morris MJ Wilson ID . The effect of heat inactivation of serum on aggregation of immunoglobulins. Immunology. (1979) 36:37–45. 422227 PMC1457381

[59] Finnie J-A Stewart RB Aston WP . A comparison of cobra venom factor-induced depletion of serum C3 in eight different strains of mice. Dev Comp Immunol. (1981) 5:697–701. doi: 10.1016/S0145-305X(81)80045-6. PMID: 6797851

[60] Haihua C Wei W Kun H Yuanli L Fei L . Cobra venom factor-induced complement depletion protects against lung ischemia reperfusion injury through alleviating blood-air barrier damage. Sci Rep. (2018) 8:10346. doi: 10.1038/s41598-018-28724-z. PMID: 29985461 PMC6037752

[61] Vogel C-W Fritzinger DC . Cobra venom factor: Structure, function, and humanization for therapeutic complement depletion. Toxicon. (2010) 56:1198–222. doi: 10.1016/j.toxicon.2010.04.007. PMID: 20417224

[62] Ricklin D Hajishengallis G Yang K Lambris JD . Complement: a key system for immune surveillance and homeostasis. Nat Immunol. (2010) 11:785–97. doi: 10.1038/ni.1923. PMID: 20720586 PMC2924908

[63] Dunkelberger JR Song W-C . Complement and its role in innate and adaptive immune responses. Cell Res. (2010) 20:34–50. doi: 10.1038/cr.2009.139. PMID: 20010915

[64] Adie EJ Kalinka S Smith L Francis MJ Marenghi A Cooper ME . A pH-sensitive fluor, CypHer^TM^ 5, used to monitor agonist-induced G protein-coupled receptor internalization in live cells. BioTechniques. (2002) 33:1152–7. doi: 10.2144/02335dd10. PMID: 12449397

[65] Pawluczkowycz AW Beurskens FJ Beum PV Lindorfer MA Van De Winkel JGJ Parren PWHI . Binding of submaximal C1q promotes complement-dependent cytotoxicity (CDC) of B cells opsonized with anti-CD20 mAbs ofatumumab (OFA) or rituximab (RTX): considerably higher levels of CDC are induced by OFA than by RTX. J Immunol. (2009) 183:749–58. doi: 10.4049/jimmunol.0900632. PMID: 19535640

[66] Van Lookeren Campagne M Wiesmann C Brown EJ . Macrophage complement receptors and pathogen clearance. Cell Microbiol. (2007) 9:2095–102. doi: 10.1111/j.1462-5822.2007.00981.x. PMID: 17590164

[67] Rivera A Siracusa MC Yap GS Gause WC . Innate cell communication kick-starts pathogen-specific immunity. Nat Immunol. (2016) 17:356–63. doi: 10.1038/ni.3375. PMID: 27002843 PMC4949486

[68] Sheu K Hoffmann A . Functional hallmarks of healthy macrophage responses: their regulatory basis and disease relevance. Annu Rev Immunol. (2022) 40:295–321. doi: 10.1146/annurev-immunol-101320-031555. PMID: 35471841 PMC10074967

[69] Kay MM. Mechanism of removal of senescent cells by human macrophages in situ. Proc Natl Acad Sci USA. (1975) 72:3521–5. doi: 10.1073/pnas.72.9.3521, PMID: 1059140 PMC433027

[70] de Back DZ Kostova EB van Kraaij M van den Berg TK van Bruggen R . Of macrophages and red blood cells; a complex love story. Front Physiol. (2014) 5:9. doi: 10.3389/fphys.2014.00009. PMID: 24523696 PMC3906564

[71] Borges MD Sesti-Costa R . Macrophages: key players in erythrocyte turnover. Hematol Transfus Cell Ther. (2022) 44:574–81. doi: 10.1016/j.htct.2022.07.002. PMID: 36117137 PMC9605915

[72] Franken L Schiwon M Kurts C . Macrophages: sentinels and regulators of the immune system. Cell Microbiol. (2016) 18:475–87. doi: 10.1111/cmi.12580. PMID: 26880038

[73] Damelang T de Taeye SW Rentenaar R Roya-Kouchaki K de Boer E Derksen NIL . The influence of human IgG subclass and allotype on complement activation. J Immunol. (2023) 211:1725–35. doi: 10.4049/jimmunol.2300307. PMID: 37843500 PMC10656437

[74] Lowell GH Smith LF Griffiss JM Brandt BL MacDermott RP . Antibody-dependent mononuclear cell-mediated antimeningococcal activity. J Clin Invest. (1980) 66:260–7. doi: 10.1172/JCI109852. PMID: 6772670 PMC371706

[75] Sharp TH Boyle AL Diebolder CA Kros A Koster AJ Gros P . Insights into IgM-mediated complement activation based on in situ structures of IgM-C1-C4b. Proc Natl Acad Sci. (2019) 116:11900–5. doi: 10.1073/pnas.1901841116. PMID: 31147461 PMC6575175

[76] Wibroe PP Helvig SY Moein Moghimi S . The role of complement in antibody therapy for infectious diseases. Microbiol Spectr. (2014) 2:10.1128/microbiolspec.aid-0015–2014. doi: 10.1128/microbiolspec.aid-0015-2014. PMID: 26105816

[77] Garson JA Quindlen EA Kornblith PL . Complement fixation by IgM and IgG autoantibodies on cultured human glial cells. J Neurosurg. (1981) 55:19–26. doi: 10.3171/jns.1981.55.1.0019. PMID: 7017080

[78] Michel M Crickx E Fattizzo B Barcellini W . Autoimmune hemolytic anemias. Nat Rev Dis Primers. (2024) 10:82. doi: 10.1038/s41572-024-00566-2. PMID: 39487134

[79] Mastellos DC Hajishengallis G Lambris JD . A guide to complement biology, pathology and therapeutic opportunity. Nat Rev Immunol. (2024) 24:118–41. doi: 10.1038/s41577-023-00926-1. PMID: 37670180

[80] Afzali B Noris M Lambrecht BN Kemper C . The state of complement in COVID-19. Nat Rev Immunol. (2022) 22:77–84. doi: 10.1038/s41577-021-00665-1. PMID: 34912108 PMC8672651

[81] Tobinai K Klein C Oya N Fingerle-Rowson G . A review of obinutuzumab (GA101), a novel type II anti-CD20 monoclonal antibody, for the treatment of patients with B-cell Malignancies. Adv Ther. (2017) 34:324–56. doi: 10.1007/s12325-016-0451-1. PMID: 28004361 PMC5331088

[82] Herter S Herting F Mundigl O Waldhauer I Weinzierl T Fauti T . Preclinical activity of the type II CD20 antibody GA101 (obinutuzumab) compared with rituximab and ofatumumab *in vitro* and in xenograft models. Mol Cancer Ther. (2013) 12:2031–42. doi: 10.1158/1535-7163.MCT-12-1182. PMID: 23873847

[83] Frischauf N Strasser J Borg EGF Labrijn AF Beurskens FJ Preiner J . Complement activation by IgG subclasses is governed by their ability to oligomerize upon antigen binding. Proc Natl Acad Sci. (2024) 121:e2406192121. doi: 10.1073/pnas.2406192121. PMID: 39436656 PMC11536094

