# Supplementary material for: Complement-mediated ADCP as a distinct and finite cytotoxic mechanism of monoclonal antibodies
Source: Front Immunol. 2026 Apr 13;17:1788948. doi: 10.3389/fimmu.2026.1788948 (PMC13111440; doi:10.3389/fimmu.2026.1788948)
Supplement: Supplementary file 1 [file Supplementaryfile1.docx]

**SUPPLEMENTAL FIGURE LEGENDS**

**Supplemental Figure 1.  Establishing complement dependency for *Fcer1g*^-/-^ BMDM ADCP.**

(**A**) Representative flow cytometry plots validating loss of FcγR surface expression in *Fcer1g*^-/-^BMDM (red) compared to WT BMDM (grey) and unstained controls (dotted line). (**B**) *Fcer1g*^-/-^BMDMs were co-cultured with thymocytes at a 10:1 T:E, 10µg/mL ⍺CD90.2, and either 10% NMS (blue) or 10% C5 depleted NMS (grey) for 12 hours. (**C**) Cumulative phagocytic index (AUC) from five independent experiments, normalized to the cADCP condition (blue). (**D**) C5 levels were quantified by ELISA across three independent batches of C5 depleted serum. These data were analyzed by two-tailed Student’s *t*-test comparisons corrections [(D) and (E)]. **P*< .05.  The error bars represent the mean ± SEM.

**Supplemental Figure 2. Representative live-cell imaging for ADCP approaching maximum**

**target capacity**

(**A to C)**Representative live-cell imaging of ADCP assays showing BMDM labeled with the cell-permeant fluorescent tracer Cell Tracker Deep Red (CTDR) co-cultured with target thymocytes at a 10:1 T:E ratio. Merged images indicate target cell proximity to labeled macrophages, while Cy5 channel images identify internalized targets as dye voids. (**A**) WT BMDM co-cultured with thymocytes, 10% NMS, and 10µg/mL αCD90.2 (fADCP + cADCP). (**B**) *Fcer1g^-/-^*BMDM were co-cultured with thymocytes, 10% NMS, and 10µg/mL αCD90.2 (cADCP). (**C**). WT BMDM co-cultured with thymocytes and 10µg/mL αCD90.2 in the absence of NMS (fADCP). Scale bar, 25 µm (A-C).

**Supplemental Figure 3. Representative live-cell imaging for cADCP at different**

**target:effector ratios.**

(**A-C**) Representative images from live-cell imaging cADCP experiments showing CTDR labeled *Fcer1g^-/-^*BMDM co-cultured with thymocytes at varying T:E ratios (10:1, 20:1, and 50:1) in the presence of 10µg/mL αCD90.2 and 10% NMS. Images were acquired during the primary cADCP challenge over 6 hours. (**A**) 10:1 T:E ratio. (**B**)  20:1 T:E ratio. (**C**) 50:1 T:E ratio. Scale bar, 25 µm (A-C).

**Supplemental Figure 4. cADCP abrogation 12hrs after primary challenge.**

**(A)**Experimental set up for B-D. *Fcer1g^-/-^*BMDM were co-cultured with thymocytes at a T:E ratios of 10:1, 20:1, or 50:1 in the presence of 10µg/mL αCD90.2 and 10% NMS for 7 hours. BMDM were then washed and allowed to recover for 12 hours prior to rechallenge with fresh thymocytes at a 10:1 T:E ratio in 10µg/mL αCD90.2 and 10% NMS for an additional 6 hours.**(B)**Rechallenge kinetics over 6 hours measured by void detection as described. **(C)** Relative cumulative phagocytic index (normalized to previously unfed controls) during rechallenge.**(D)** Relative void max (normalized to previously unfed controls) during rechallenge. Data shown are mean ± SEM for three independent experiments (B-D). Data were analyzed using an ordinary one-way ANOVA with Dunnett multiple comparisons correction [(C) and (D)]. **P*< .05
