# Supplementary figures and images for: Complement-mediated ADCP as a distinct and finite cytotoxic mechanism of monoclonal antibodies
Source: Front Immunol. 2026 Apr 13;17:1788948. doi: 10.3389/fimmu.2026.1788948 (PMC13111440; doi:10.3389/fimmu.2026.1788948)

**A**

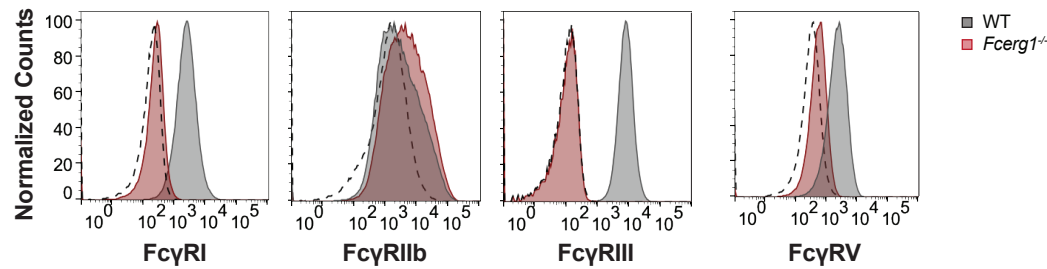

**B**

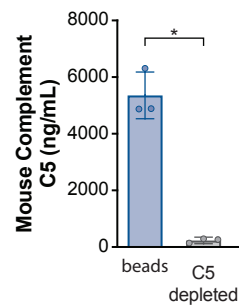

**C**

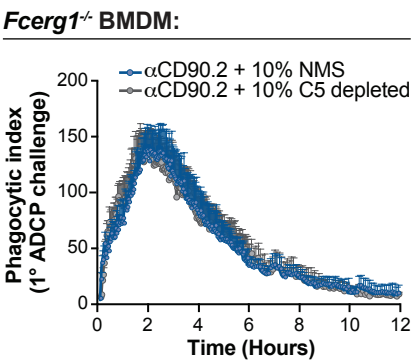

**D**

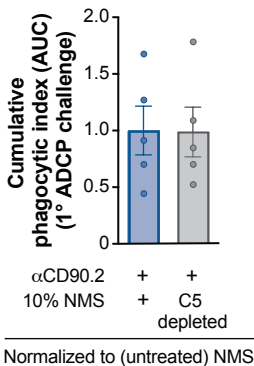

Supplement: Supplementary file 2 [file Supplementaryfile2.pdf]

**A**

Wildtype BMDM +  $\alpha$ CD90.2:

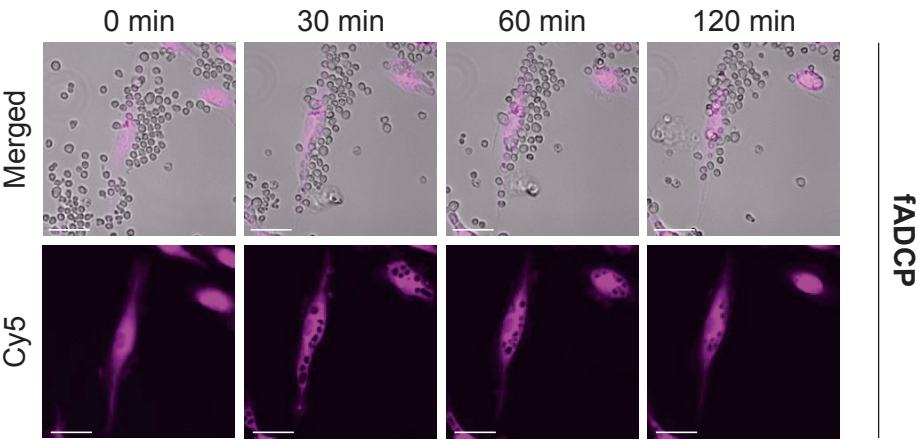

**B**

*Fcer1g*<sup>-/-</sup> BMDM +  $\alpha$ CD90.2 + NMS:

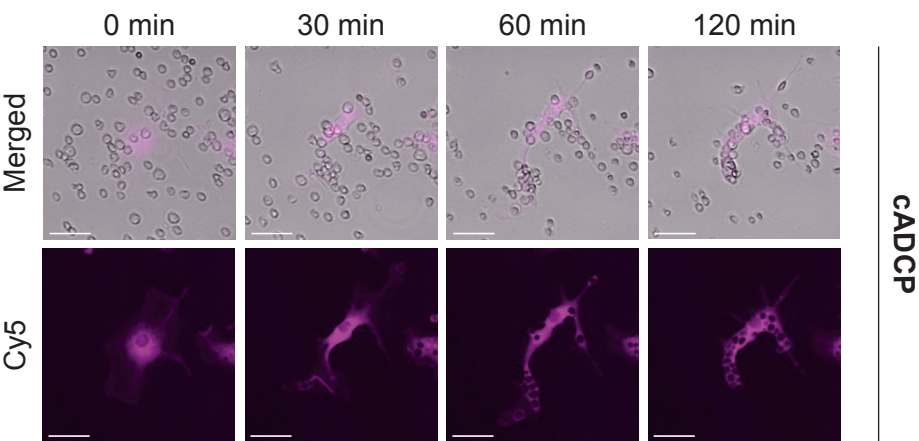

**C**

Wildtype BMDM +  $\alpha$ CD90.2 + NMS:

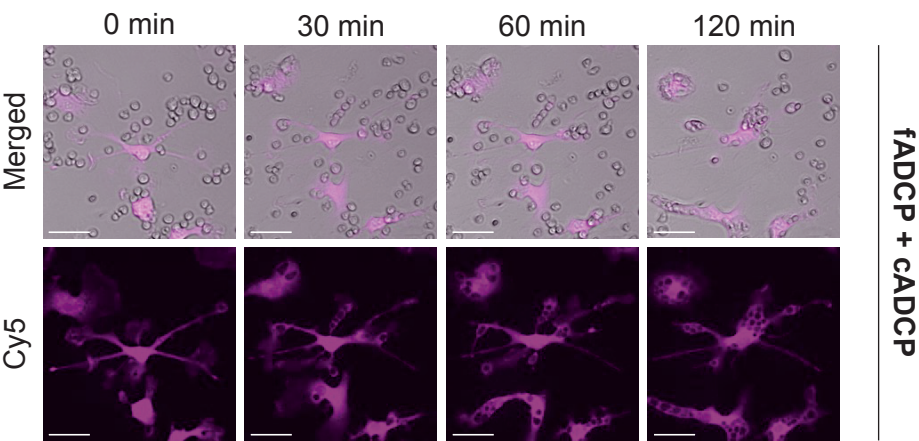

Supplement: Supplementary file 3 [file Supplementaryfile3.pdf]

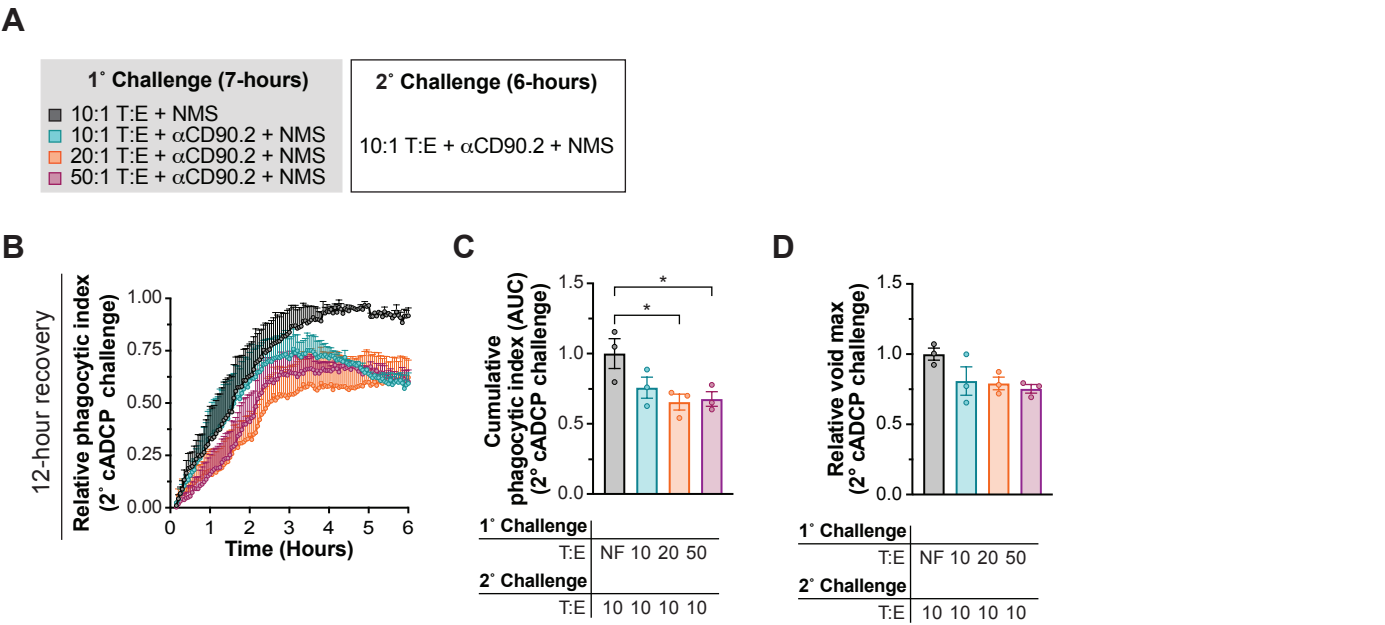

Supplement: Supplementary file 5 [file Supplementaryfile5.pdf]
