# Supplementary material for: Complement-mediated ADCP as a distinct and finite cytotoxic mechanism of monoclonal antibodies
Source: Front Immunol. 2026 Apr 13;17:1788948. doi: 10.3389/fimmu.2026.1788948 (PMC13111440; doi:10.3389/fimmu.2026.1788948)

A

10:1 E:T - *Fcer1g*$^{-/-}$ BMDM + αCD90.2 + NMS:

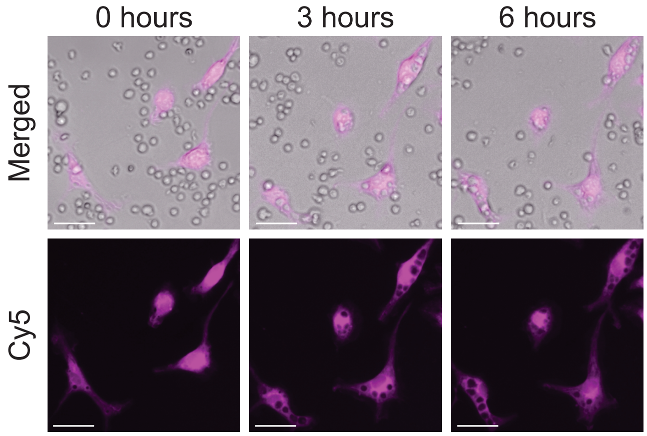

B

20:1 T:E - *Fcer1g*$^{-/-}$ BMDM + αCD90.2 + NMS:

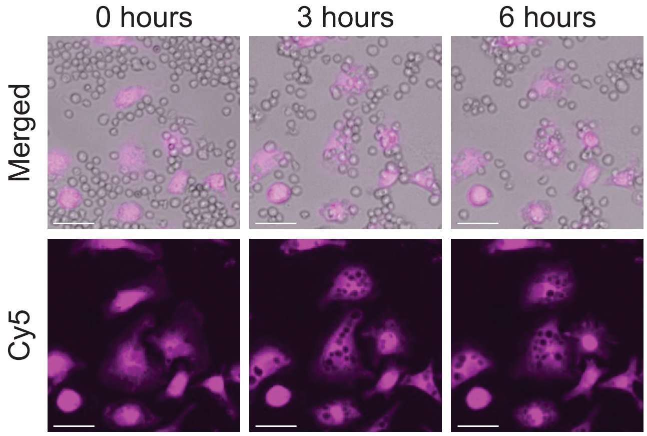

C

50:1 - *Fcer1g*$^{-/-}$ BMDM + αCD90.2 + NMS:

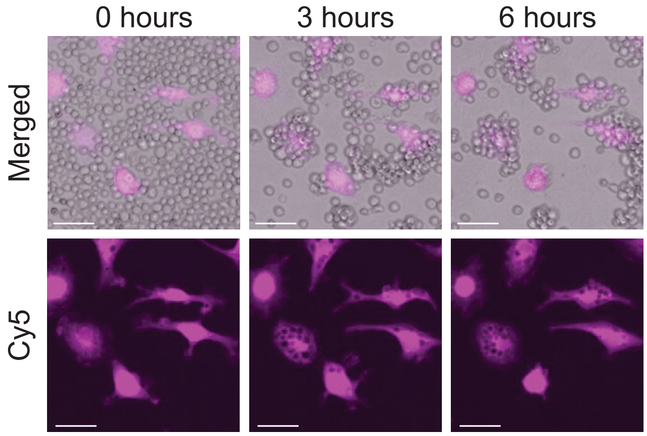

Supplement: Supplementary file 4 [file Supplementaryfile4.pdf]
